# Novel Detection of Atmospheric Turbulence Profile Using Mie-Scattering Lidar Based on Non-Kolmogorov Turbulence Theory

**DOI:** 10.3390/e25030477

**Published:** 2023-03-09

**Authors:** Jiandong Mao, Yingnan Zhang, Juan Li, Xin Gong, Hu Zhao, Zhimin Rao

**Affiliations:** 1 School of Electrical and Information Engineering, North Minzu University, North Wenchang Road, Yinchuan 750021, China; 20207206@stu.nun.edu.cn (Y.Z.);; 2 Key Laboratory of Atmospheric Environment Remote Sensing of Ningxia Province, North Wenchang Road, Yinchuan 750021, China

**Keywords:** non-Kolmogorov turbulence, Mie-scattering lidar, refractive index structure constant, probability density distribution, residual turbulent scintillation theory

## Abstract

Turbulence can cause effects such as light intensity fluctuations and phase fluctuations when a laser is transmitted in the atmosphere, which has serious impacts on a number of optical engineering application effects and on climate improvement. Therefore, accurately obtaining real-time turbulence intensity information using lidar-active remote sensing technology is of great significance. In this paper, based on residual turbulent scintillation theory, a Mie-scattering lidar method was developed to detect atmospheric turbulence intensity. By extracting light intensity fluctuation information from a Mie-scattering lidar return signal, the atmospheric refractive index structure constant, $C_{n}^{2}$, representing the atmospheric turbulence intensity, could be obtained. Specifically, the scintillation effect on the detection path was analyzed, and the probability density distribution of the light intensity of the Mie-scattering lidar return signal was studied. It was verified that the probability density of logarithmic light intensity basically follows a normal distribution under weak fluctuation conditions. The $C_{n}^{2}$ profile based on Kolmogorov turbulence theory was retrieved using a layered, iterative method through the scintillation index. The method for detecting Kolmogorov turbulence intensity was applied to the detection of the non-Kolmogorov turbulence intensity. Through detection using the scintillation index, the corresponding ${\tilde{C}}_{n}^{2}$ profile could be calculated. The detection of the ${\tilde{C}}_{n}^{2}$ and $C_{n}^{2}$ profiles were compared with the Hufnagel–Valley (HV) night model in the Yinchuan area. The results show that the detection results are consistent with the overall change trend of the model. In general, it is feasible to detect a non-Kolmogorov turbulence profile using Mie-scattering lidar.

## 1. Introduction

Atmospheric turbulence is one of the most prominent features in the atmospheric boundary layer [1]. The main causes of atmospheric turbulence are solar radiation, wind shear, and heat convection, which lead to a change in the temperature field and velocity field [2]. Atmospheric turbulence will cause random fluctuations in the atmospheric refractive index. The Earth’s atmosphere is in a state of turbulent motion all the time, and the turbulence phenomenon is closely related to astronomy, meteorology, aviation, and other fields.

In recent years, lasers have been widely used in communication and remote sensing, which has also greatly promoted research into light wave transmission in turbulent atmospheres. When light waves are transmitted in the atmosphere, the relevant features are affected by atmospheric turbulence and show an irregular changing state [3]. The randomness of atmospheric turbulence, which makes the refractive index of the atmosphere appear in random fluctuations, results in an optical turbulence effect in the transmission of light waves in the atmosphere [4]. This mainly includes light intensity fluctuations, arrival angle fluctuations, spot drifts, beam expansions, etc., which all lead to laser wavefront distortions and coherence damage, resulting in some difficulties in atmospheric remote sensing and satellite communication [5,6,7,8]. At the same time, the atmospheric turbulence movement is accompanied by the transfer and exchange of energy, momentum, and matter, which will have an impact on temperature and humidity. In some arid areas, the atmospheric turbulence effect will even directly affect the amount of rainfall, having a serious impact on the climate [9].

The physical quantity measurements commonly used to describe atmospheric turbulence effects include the scintillation index, the refractive index structure constant, and coherence length [10,11,12]. Among them, the atmospheric refractive index structure constant $C_{n}^{2}$ is an important physical quantity characteristic that can represent atmospheric turbulence intensity and directly describes the degree of variation in refractive index uneven random fluctuations. The distribution profile of the atmospheric refractive index structure constant with height is called the atmospheric turbulence profile. The acquisition of the atmospheric turbulence profile is the basis for the study of the vertical distribution characteristics of turbulence intensity, along with the statistics regarding the temporal and spatial changes in turbulence. Therefore, the accurate detection of the basic characteristics of the spatial and temporal structure of atmospheric turbulence and the acquisition of the real-time distribution of atmospheric turbulence intensity is of great significance for the evaluation of atmospheric laser transmission, optical communication, and the meteorological prediction model [13,14]. 

Lidar, as an active remote-sensing technology, uses a laser as the light source and obtains atmospheric distribution information along the laser beam transmission path by detecting the backscattering signals of the interaction between the laser and the atmosphere. At present, the lidar technologies commonly used to detect atmospheric turbulence mainly include differential image motion lidar (DIM), differential column image motion lidar (DCIM), and differential wavefront lidar (DW). DIM lidar is designed according to the principle of differential image motion measurement (DIMM). The atmospheric coherence length $r_{0}$ is obtained by receiving the dither variance of the centroid of the spot on the aperture. In 2016, Zhou et al. developed an atmospheric turbulence profile lidar based on differential image motion and conducted some experiments, but its resolution was limited [15]. In recent years, the S-DIMM+ method was proposed on the basis of DIMM, and the Shack–Hartmann wavefront sensor with a wide field of view was used to detect the atmospheric turbulence profile [16,17]. In 2018, Wang et al. proposed an improved S-DIMM+ method that clearly shows the location of strong turbulent layers [18]. In 2021, Kovadlo et al. determined the relationship between the height of the atmospheric turbulence layer and the angular displacement by measuring the image jitter using the S-DIMM+ method [19]. DICM lidar uses a charge-coupled device (CCD) image plane to image two scattered light columns to obtain the coherent length and turbulence profile of the atmosphere. In 2014, Tang et al. studied the detection performance of heterodyne lidar for turbulence [20]. DW lidar can invert the atmospheric turbulence profile by calculating the variance in the wavefront jitter of two light spots on the intensified charge-coupled device (ICCD). In 2021, Wang et al. simulated and experimentally verified the performance of differential wavefront lidar in detecting atmospheric turbulence [21]. The above lidar basically uses the dither variance of the image light spot to detect the atmospheric turbulence profile.

Mie-scattering lidar is an atmospheric lidar system designed based on the Mie-scattering theory proposed by Gustav Mie [22]. Compared with the other lidar methods mentioned above, Mie-scattering lidar is smaller in size, simple in operation, cost-saving, and easy to move, with high spatial and temporal resolution and a strong return signal. It is generally used to measure the height of cloud bases, aerosol structures, or atmospheric visibility below 30 km. In the method proposed in this paper, due to the presence of turbulence, there are certain fluctuations in the optical power of the echo wave detected by Mie-scattering lidar, including aerosol particle fluctuations and turbulence fluctuations. By adding a small iris diaphragm to the telescope, the fluctuation information caused by turbulence can be extracted from the Mie-scattering lidar return signal, and the atmospheric turbulence can be detected by the intensity fluctuation in the return signal. $C_{n}^{2}$ is a parameter that directly describes atmospheric turbulence; for Mie-scattering lidar, the $C_{n}^{2}$ profile can be obtained from return signal fluctuations. This is different from the other lidar detection principles mentioned above.

The Kolmogorov turbulence theory is a local isotropic turbulence theory, which is an idealized theory. It is widely used in the inversion of turbulence intensity in the process of detecting atmospheric turbulence with lidar. However, some experiment results show that the turbulence characteristics of the tropopause and the stratosphere are significantly different when using Kolmogorov theory [23,24]. In this case, the turbulence is no longer uniform in three dimensions, so the Kolmogorov theory is no longer applicable. Therefore, it is necessary to study non-Kolmogorov turbulence profile theory, which is closer to the actual situation, to describe the actual turbulence state [25]. In 2018, Toselli et al. analyzed the light intensity distribution, beam propagation, and scintillation index of non-Kolmogorov turbulence in a double-passage lidar system through theoretical and simulation methods [26]. Therefore, this paper proposes a method to detect the intensity of non-Kolmogorov turbulence using Mie-scattering lidar.

In this paper, based on residual turbulent scintillation (RTS) theory, a Mie-scattering lidar method suitable for measuring the atmospheric turbulence profile is proposed. The scintillation index of different heights was obtained by calculating the fluctuation in light intensity, and the atmospheric turbulence profile was calculated. Some experiments were performed. The turbulence theory was extended from the Kolmogorov theory to the non-Kolmogorov theory, and the atmospheric turbulence profiles under the two theories were obtained and compared with the $C_{n}^{2}$ theoretical model of the Yinchuan region. The results show that it is feasible to use Mie-scattering lidar to detect non-Kolmogorov atmospheric turbulence.

## 2. Principle of Detection

### 2.1. Residual Turbulent Scintillation Theory

The detection principle of Mie-scattering lidar is based on the residual turbulent scintillation theory. When light waves are transmitted in the atmosphere, they will interact with atmospheric molecules and aerosols, and the subsequent scattered signals will be received by Mie-scattering lidar. Due to the existence of turbulence, the received return signal fluctuates. According to the residual turbulent scintillation theory, the signal fluctuation caused by aerosols can be smoothed out by adding a small iris diaphragm to the telescope, retaining only the signal fluctuation information caused by turbulence to measure the atmospheric turbulence intensity. The residual turbulent scintillation theory and its limitations are introduced below.

Belen’kii et al. proposed the RTS theory for measuring atmospheric turbulence, expressed as when the light propagates in the atmosphere, if the radius, $a_{r}$, of the scatterer is smaller than the spatial correlation scale, $l_{I}$, of the light intensity fluctuation (where $l_{I}=\sqrt{\lambda L}$, λ is the wavelength of the incident laser and L is the length of the detection path) for any received aperture, D, the backscattered light intensity signal can weaken the average aperture effect, and the light intensity fluctuation will exist [27].

Under the following four conditions:

(1) Single scattering approximation and uniform aerosol particle size distribution, average path length L, aerosol particle radius $\bar{a}$, with the incident laser wavelength λ meeting ≫ $L\gg {\bar{a}}^{2}/\lambda$;

(2) Atmospheric optical thickness: τ≪1;

(3) The relevant scale of radial light intensity $l_{I\|}$($l_{I\|}\approx L$) is far more than the scale of the scatterer $c\tau_{i}/2$, and the scatterer scale is far more than the laser wavelength $L\gg c\tau_{i}/2\gg \lambda$ (c is the speed of light and $\tau_{i}$ is the pulse width);

(4) Under the condition of weak fluctuations ($\sigma_{i}^{2}\ll 0.6$), the telescope receiving aperture D and the turbulent coherence length of a plane wave $\rho_{0}$, $l_{I}$, and $\rho_{cv}$ meet $D\gg \rho_{0}\gg l_{I}\gg \rho_{cv}$, where $\sigma_{i}^{2}$ is the variance of logarithmic light intensity scintillation, $\rho_{cv}=2L/ka_{ef}$ is given by the van Cittert–Zernike theorem, $a_{ef}$ is the effective beam size at the distance L from the light source, and k=2π/λ is the wave number.

The intensity scintillation of backscattered light has two spot scales: the aerosol spot scale ($l_{a}=\lambda F/D$) affected by aerosols; and the turbulence spot scale ($l_{t}=Fl_{I}/L$), where F is the focal length of the telescope. As $l_{t}/l_{a}=D/\sqrt{\lambda L}$, and, in general, $D\gg \sqrt{\lambda L}$ in the actual large diameter receiving system, then $l_{t}\gg l_{a}$ is generally met.

Since the scintillation spot has two different spatial scales, the fluctuation in backscattering light intensity can be obtained by changing the size of the aperture $d_{0}$ of the telescope’s field of view:

(1) $$\{\begin{array}{c} \sigma_{P}^{2}=1+2\sigma_{I}^{2},\;d_{0}\ll l_{a}\\ \sigma_{P}^{2}=\sigma_{I}^{2},\;l_{a}\ll d_{0}\ll l_{t}\\ \sigma_{P}^{2}=O\left(\sigma_{I}^{2}/\left(d_{0}/F\sqrt{\lambda /L}\right)\right),\;l_{t}\ll d_{0} \end{array}$$

where $\sigma_{P}^{2}$ is the variance in the normalized intensity fluctuation in backscattering light passing through an aperture, and $\sigma_{I}^{2}$ is the variance in the normalized intensity fluctuation in the beam. In Equation (1), the first term indicates that the variance in light intensity fluctuations includes aerosol speckle fluctuations and turbulent fluctuations; the second term indicates that the aperture smooths the aerosol speckle fluctuations but includes turbulent fluctuations, and the third term indicates that the aerosol and turbulent fluctuations are smoothed by the aperture, and the total received energy does not fluctuate. Therefore, the atmospheric turbulence fluctuation information along the laser beam propagation path can be obtained by receiving the light intensity fluctuation measurement data, then the atmospheric turbulence profile can be obtained.

According to RTS theory, when measuring the atmospheric turbulence profile, the parameters directly related to lidar include the laser incident wavelength, λ, telescope aperture, D, telescope focal length, F, telescope aperture, $d_{0}$, and lidar detection distance, L.

### 2.2. Design of Mie-Scattering Lidar

**Figure 1:**
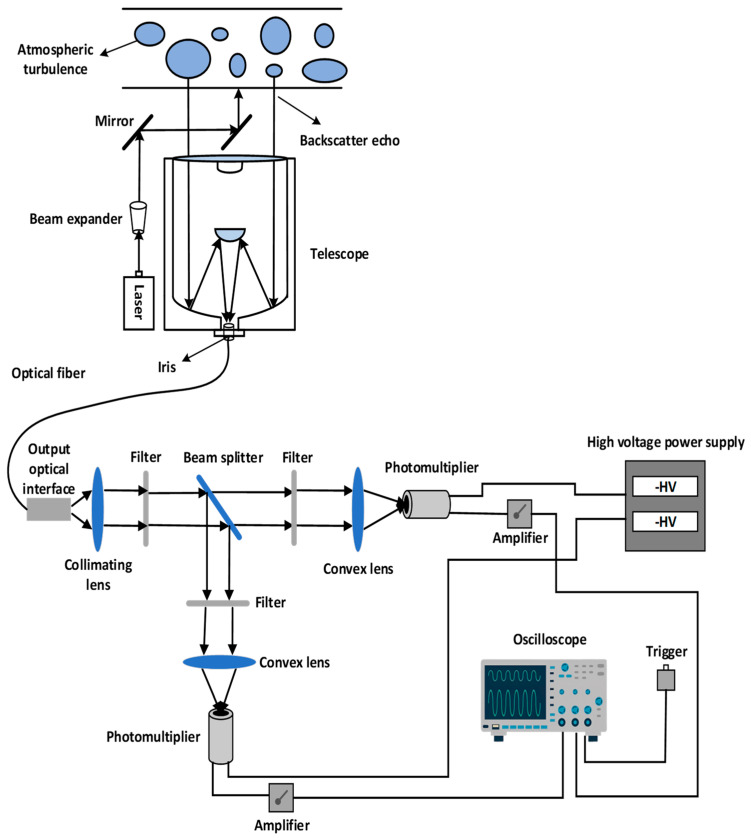
Schematic diagram of the Mie-scattering lidar system.

The Mie-scattering lidar system used to detect atmospheric turbulence consists of a laser-emitting system, a receiving system to receive the return signals generated by the scattering effect of atmospheric molecules and aerosol particles, and the atmospheric turbulence effect, a spectroscope system to divide and filter the return signals, and a data acquisition system to amplify and acquired return signals. Figure 1 shows a schematic diagram of the Mie-scattering lidar system.

The laser beam is emitted by an Nd:YAG laser in the transmitting system. After expanding through the beam expander, the laser beam is transmitted to the detection height through atmospheric Fresnel diffraction, atmospheric molecular extinction, and atmospheric turbulence. The backscattering return signal is received by a Schmidt-Cassegrain telescope and is transmitted through the small aperture variable diaphragm and optical fiber into the spectroscope system. The signal is then collected and processed by the data acquisition system and transmitted to the computer.

For the emitting system, the laser used in this study is an Nd:YAG solid-state laser, which consists of an incident laser with a fundamental frequency wavelength of 1064 nm and a double frequency wavelength of 532 nm. In this study, the incident wavelength is selected as 532 nm, the corresponding single-pulse energy is 150 mJ, the energy stability is less than 3%, the power stability is less than 2%, and the repetition frequency is 10 Hz.

For the receiving system, according to the backscattering multiplier effect of lidar light waves in the nonuniform medium, the effect is most obvious when the combined transceiver structure is adopted [28,29]. Therefore, the coaxial structure of light intensity glinting lidar is adopted, which has the characteristics of a small blind area, convenient collimation, and a compact structure. The telescope used in this study is a Schmidt-Cassegrain reversion type, with a parabolic primary mirror and two hyperboloid secondary mirrors in the barrel. The telescope aperture D  = 254 mm, and the focal length F  = 2500 mm.

In the spectroscope system, the laser signal transmitted from the optical fiber is collimated through a flat, convex lens and then passes through a 532 nm filter to filter the stray light. The beam splitter with R:T = 3:7 is used to divide the beam in two ways: the reflected signal passes through a filter with an optical density of 0.1 to the flat, convex lens and then converges into the photomultiplier (PMT1) and the transmitted signal passes through a filter with an optical density of 0.2 to the flat, convex lens and then converges into the PMT2. The purpose of using different optical density filters is to test the built Mie-scattering lidar system and to judge the amplification of the strong and weak signals.

For the data acquisition system, the PMT selected in this study and the high-voltage power supply were produced by Licel in Germany and can convert weak optical signals into electrical signals. In order to facilitate the collection and display of the PMT-converted voltage signal by the data acquisition unit, an amplifier is installed to amplify the electrical signal and eliminate the signal oscillation. The data acquisition unit is a high-speed fluorescent oscilloscope, which has a bandwidth of 1 GHz, a sampling frequency of 100 M/s, and a recording length of 10,000 points and can sample the data points after 512 times that of the average return signal. Table 1 specifically lists the main parameters of atmospheric turbulence detection using Mie-scattering lidar.

### 2.3. Detection Method

According to the RTS theory described above, the spatial correlation scale $l_{I}$ of the light intensity fluctuation is calculated. According to the formula $l_{I}=\sqrt{\lambda L}$, the spatial correlation scale increases with the increase in detection distance and incident wavelength. Here, the wavelength of the incident laser is determined to be 532 nm. When the maximum detection distance is 3 km, the maximum spatial correlation scale, $l_{I}$, is approximately 39.9 mm. The telescope aperture D = 254 mm obviously meets the requirements of a large aperture receiving system $D\gg \sqrt{\lambda L}$.

According to the calculations, the aerosol spot scale $l_{a\;}$ = 5.236 μm. When the detection distance L  = 1~3 km, the turbulence-related scale $l_{t}$ is in the range of 0.058~0.033 mm. If we want to satisfy the condition $l_{a}\ll d_{0}\ll l_{t}$, then $d_{0}<$0.03 mm. Where $d_{0}$ is the diameter of the iris diaphragm. The size of the iris diaphragm is too small and difficult to implement in manufacturing. Increasing the focal length F and shortening the detection distance L can increase the iris diaphragm diameter. However, the long focal length and small iris diaphragm design will directly lead to a smaller field of view, a received weakened return signal, an increased blind area and transition area, and an increase in difficulty when aligning the optical axis and machining the aperture. Therefore, it is necessary to find a balance point in line with or close to the principle. When the condition $l_{a}\ll d_{0}\ll l_{t}$ cannot be strictly satisfied, ensure that $d_{0}$ is close to $l_{t}$. Here, after considering the operability of the experiment and the actual conditions of the laboratory equipment, the diameter $d_{0}\geq$ 0.5 mm is selected for the iris diaphragm.

In this study, light intensity fluctuation is used to detect atmospheric turbulence. The fluctuation in light intensity on the receiving plane is called scintillation, which is the most important effect of turbulent atmospheric transmission. The scintillation index is used to describe the intensity of light intensity fluctuations, namely the normalized variance of light intensity fluctuations:

(2) $$\beta_{I}^{2}=\frac{\langle I^{2}\rangle -{\langle I\rangle }^{2}}{{\langle I\rangle }^{2}}$$

where I is the received light intensity, and 〈·〉 represents the ensemble average. 

Under a weak fluctuation condition $\beta_{I}^{2}<$ 1, the relationship between the scintillation index and logarithmic amplitude fluctuation variance $\sigma_{\chi }^{2}$ is as follows:

(3) $$\beta_{I}^{2}=\sigma_{lnI}^{2}=\exp\left(4\sigma_{\chi }^{2}\right)-1\approx 4\sigma_{\chi }^{2}$$

Since the weak fluctuation condition is always satisfied in the actual detection process of Mie-scattering lidar, the emitted Gaussian beam can be approximately regarded as a spherical wave, and most propagation problems meet the conditions of the turbulent inertia region [2]. Under the Kolmogorov turbulent power spectrum, for the propagation process from z = 0 to z = L, the logarithmic amplitude fluctuation variance in the spherical wave is calculated by

(4) $$\sigma_{\chi }^{2}\left(L\right)=0.5631k^{7/6}\int_{0}^{L}C_{n}^{2}\left(z\right){[\frac{z\left(L-z\right)}{L}]}^{5/6}dz$$

where k is the wave number and k=2π/λ, $C_{n}^{2}$ is the refraction index structure constant of the atmosphere.

On the vertical detection path, the scintillation index of the spherical wave is written as

(5) $$\beta_{I}^{2}\left(L\right)=4\sigma_{\chi }^{2}=2.25k^{7/6}\int_{0}^{L}C_{n}^{2}\left(z\right){\left[\frac{z\left(L-z\right)}{L}\right]}^{5/6}dz$$

In summary, the atmospheric turbulence detection method adopted is as follows: based on RTS theory, first, the scintillation index $\beta_{I}^{2}$ at each distance is obtained according to Equation (2); then, the variation trend of $\beta_{I}^{2}$ and $C_{n}^{2}$ along the propagation path is obtained according to Equation (5), and finally, the atmospheric turbulence profile can be obtained. Before the experiment, we carried out some systematic simulation studies to ensure the feasibility of the experimental scheme and the selection of system parameters [30,31].

**Figure 2:**
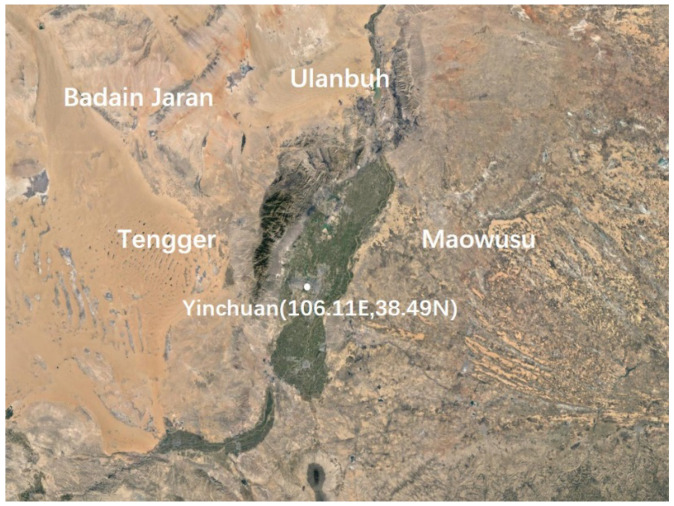
Geographical location map of Yinchuan.

Yinchuan is located in northwest China in the central and northern parts of the Ningxia Plain, surrounded by the Yellow River in the east and the Helan Mountains in the west. It is also surrounded by the Badain Jaran, Ulanbuh, Tengger, and Maowusu deserts and has a typical temperate continental semi-arid climate, as shown in Figure 2. Its geographical co-ordinates are 35°14′–39°23′ north latitude and 104°17′–107°39′ east longitude. According to the long-term observation of aerosol particle size distribution, the average aerosol particle size is usually $\bar{a}=$1.0 μm [32]; however, in spring, the aerosol particle size is slightly larger than average, but from an order of magnitude perspective, the conditions of $L\gg {\bar{a}}^{2}/\lambda$ are clearly met.

## 3. Experimental Results and Analysis

### 3.1. Light Intensity Distribution

Random fluctuations in the atmospheric refractive index lead to random fluctuations in laser beam intensity. As a specific random process, light intensity fluctuation can be quantitatively described by the probability density distribution of light intensity, and the probability density distribution is also the most basic method to describe the statistical characteristics of light intensity fluctuation. The working principle of Mie-scattering lidar is to obtain the scintillation index of the beam by analyzing the intensity fluctuation in the received return signal and then obtain the atmospheric turbulence intensity information by inversion. Therefore, it is of great significance to study the statistical characteristics of the light intensity fluctuation in the return signal to detect atmospheric turbulence using Mie-scattering lidar and to improve the working performance of the lidar.

A large number of theoretical and experimental studies have shown that, under weak turbulence fluctuation conditions, the probability density distribution of signal light intensity fluctuations meets the log-normal distribution; that is, the logarithmic intensity follows the normal distribution [33]. In order to verify whether the Mie-scattering lidar method adopted in this study meets this conclusion, a statistical analysis of the light intensity fluctuations in the return signal received via Mie-scattering lidar in the vertical detection direction was carried out, and the actual probability distribution of light intensity was fitted.

The built Mie-scattering lidar is used to measure turbulence in the vertical direction. For the vertical detection path, the atmospheric turbulence intensity varies with height, so obtaining the probability density distribution of the back-wave light intensity signal can provide a theoretical basis for the inversion of the atmospheric refractive index structure constant.

The wavelength of the laser source is 532 nm, and the emission frequency is 10 Hz. As the probability density distribution of light intensity is a statistical feature of light intensity fluctuation, a large sample size is required. In this paper, 10,000 sample points composed of 10,000 pulses were analyzed as a group. By taking the 1.5 km transmission path as an example, the probability distribution characteristics of the light intensity received via Mie-scattering lidar were analyzed.

**Figure 3:**
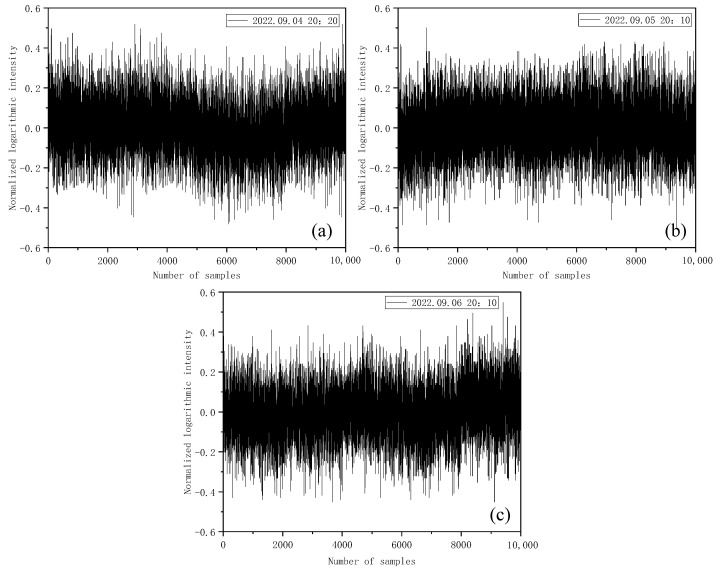
Normalized logarithmic intensity of Mie-scattering lidar return signal. (**a**) Normalized logarithmic intensity of return signal at 20:20 on 4 September 2022. (**b**) Normalized logarithmic intensity of return signal at 20:10 on 5 September 2022. (**c**) Normalized logarithmic intensity of return signal at 20:10 on 6 September 2022.

First, the data collected in the experiment were normalized to make the average logarithmic light intensity (after normalization) 0, that is, <lnI> = 0. The signal collected by Mie-scattering lidar is the electrical signal after photoelectric conversion, which has a linear relationship with the light intensity signal. Therefore, the optical signal can be replaced by the electrical signal characteristics, and the statistical characteristics of the optical signal can be obtained by analyzing the electrical signal. The specific normalization method is as follows: after taking the logarithm, calculate the average value of the original light intensity data; then, subtract the minimum value from the maximum value of the log light intensity, and finally, subtract the average value from the log light intensity and divide by the difference between the maximum value and the minimum value to obtain the normalized log light intensity with a mean value of 0. Here, the data from three days were actually tested and analyzed respectively. Figure 3 shows the normalized logarithmic light intensity fluctuation in the Mie-scattering lidar return signal obtained. 

**Figure 4:**
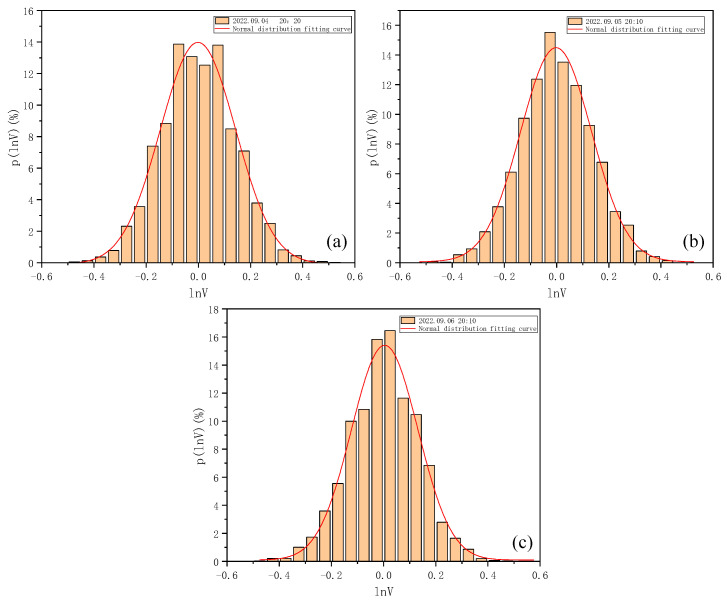
Histogram of probability density distribution of normalized logarithmic light intensity and its fitted results. (**a**) Histogram of probability density distribution of normalized logarithmic light intensity and its fitted results at 20:20 on 4 September 2022. (**b**) Histogram of probability density distribution of normalized logarithmic light intensity and its fitted results at 20:10 on 5 September 2022. (**c**) Histogram of probability density distribution of normalized logarithmic light intensity and its fitted results at 20:10 on 6 September 2022.

As can be seen from Figure 3, the logarithmic light intensity signal fluctuates around the mean value with different intensities. According to Equation (2), the scintillation index of the three groups of data can be obtained as 0.000021, 0.000017, and 0.000019, respectively. The probability density distribution statistics of the return signal intensity signals of each group of data were determined, and the normalized logarithmic light intensity data were divided into different intervals for the statistics to obtain the normalized data frequency; thus, further obtaining the probability density distribution histogram and its fitted results, as shown in Figure 4. 

It can be seen from Figure 4 that the log-normal distribution can well describe the actual shape of the probability density distribution of the logarithmic light intensity of the received return power in the actual detection of atmospheric turbulence by Mie-scattering lidar; that is, it verifies that, under the weak fluctuations in Mie-scattering lidar, the return logarithmic light intensity basically follows the log-normal distribution.

### 3.2. Detection of Atmospheric Turbulence

#### 3.2.1. Kolmogorov Turbulence Detection

As mentioned above, the scintillation index $\beta_{I}^{2}$ is received by Mie-scattering lidar when detecting atmospheric turbulence is the spherical wave scintillation index caused by the turbulence effect of the transmitting path. First, the scintillation effect and turbulence profile described by the Kolmogorov theory were detected and analyzed.

The experiment was conducted from August to September 2022, and the observation lasted for approximately 20 days. Most of the time, the weather was clear and the visibility was high, and there were no obvious clouds in the detection path. Due to the strong sky background light noise received via lidar during the day, the observation was only conducted after 20:00 LST (local standard time) every night, lasting until 22:00 LST, for two hours a day.

**Figure 5:**
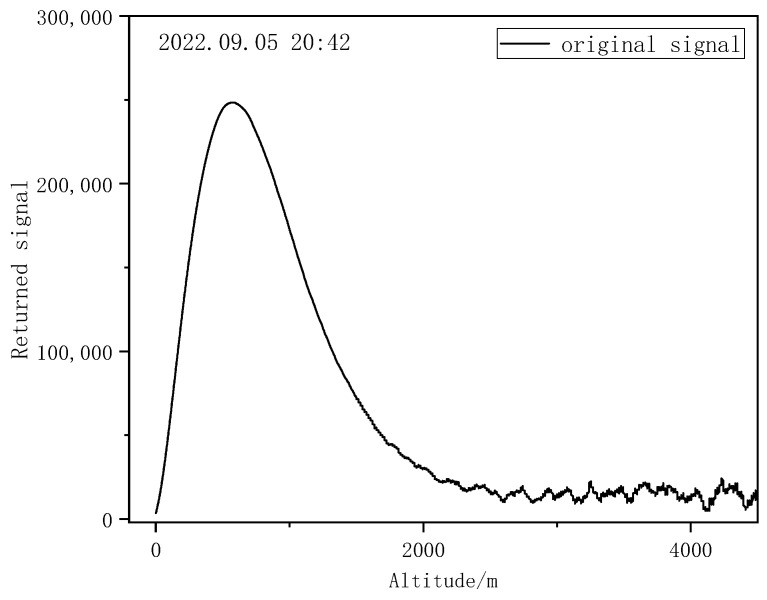
Variation in Mie-scattering lidar return signal with height.

A high-speed fluorescent oscilloscope was used to collect the return signal received via lidar. Figure 5 shows the variation in return signal at a height in the range of 0~4.5 km with a single pulse and a height resolution of 37.5 m.

The change in scintillation index in the vertical transmission path was calculated using the collected Mie-scattering lidar return signal. In the vertical direction, the turbulence intensity of the far-field signal is very weak. In the process of laser transmission, the amount of light intensity flicker caused by the turbulence of far-field signals will be relatively small. Moreover, the background sky light noise, granular noise, amplifier noise, and other noise will cause a fluctuation in light intensity changes, and the calculated scintillation index is not only caused by turbulence changes. Therefore, vertical detection by Mie-scattering lidar requires a high signal-to-noise ratio to obtain an accurate far-field signal. In this study, the Mie-scattering lidar return signal was denoised by the sliding window average method to some extent. As can be seen from Figure 5, the return signal of the Mie-scattering lidar method used in the experiment has a low signal-to-noise ratio after a height of 2500 m. Due to the lidar field of view overlap below 600 m to ensure detection accuracy, after careful consideration, the data within the 600~2000 m height range were analyzed in this study.

Usually, the scintillation index $\beta_{I}^{2}$ is calculated by Equation (2). In practice, the measured value is the voltage signal, and the signal voltage U is directly proportional to the incident signal light intensity I, so Equation (2) can be transformed into voltage form [34]:

(6) $$\beta_{I}^{2}=\frac{\sigma_{U}^{2}}{{\langle U\rangle }^{2}}=\frac{\langle U^{2}\rangle -{\langle U\rangle }^{2}}{{\langle U\rangle }^{2}}$$

Therefore, the scintillation index is calculated by measuring the mean <U> and the variance $\sigma_{U}^{2}$ of the signal voltage and by substituting them into Equation (6).

**Figure 6:**
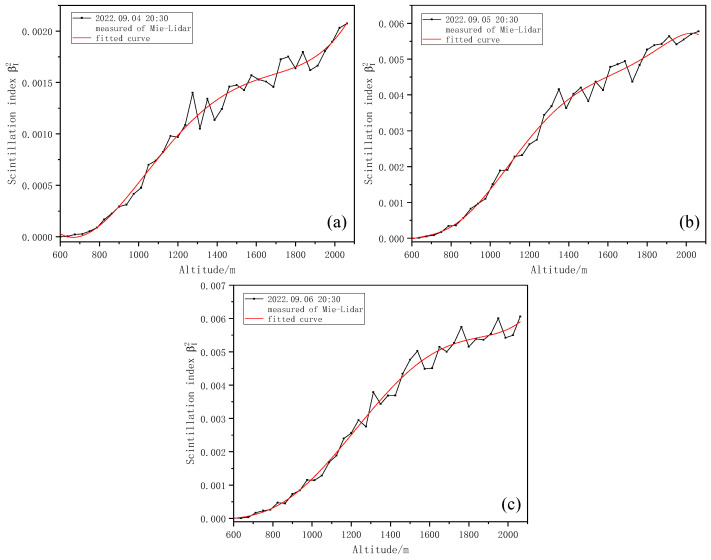
Variation in scintillation index with height. (**a**) Variation in scintillation index with height at 20:30 on 4 September 2022. (**b**) Variation in scintillation index with height at 20:30 on 5 September 2022. (**c**) Variation in scintillation index with height at 20:30 on 6 September 2022.

The scintillation index of different heights was calculated by the return signal. Figure 6 shows the variation in the scintillation index with height over three different time periods, and the height resolution is 37.5 m. The red line refers to the fitted curve of the detected scintillation index. In the process of calculating the scintillation index, due to the existence of the lidar overlap area and considering that the normalization should be carried out at the place where the return signal is the strongest, according to the research and analysis of multiple groups of measured data, the normalized height was finally specified as 600 m, and the diameter of iris diaphragm of the telescope was selected as $d_{0}$ = 0.5 mm.

As can be seen from Figure 6, the scintillation index basically conforms to the trend of gradually increasing with the increase in height.

In this study, the scintillation index profile was used to obtain the atmospheric refractive index constant profile $C_{n}^{2}\left(h\right)$. The influence of internal and external scales of atmospheric turbulence was not considered, and the power spectrum of atmospheric turbulence is the Kolmogorov spectrum. Therefore, on the vertical detection path, the scintillation index was calculated by Equation (5), and the Gaussian beam emitted by lidar is regarded, approximately, as a spherical wave.

In this study, the Newton iteration method is used to invert the $C_{n}^{2}$ profile. The specific operation is as follows: first, $C_{n}^{2}$ is divided into n segments in the whole height range of 0~L, and the corresponding interval of each segment is Δh. Second, with the idea of stratification, assuming that $C_{n}^{2}$ in the height of each segment is the same constant, $C_{n}^{2}$ can be extracted from Equation (5) when calculating the turbulence intensity in each segment, and then $C_{n}^{2}$ in the 0~Δh interval can be obtained, namely $C_{n1}^{2}$. Third, when combined with $C_{n1}^{2}$ and using the iterative algorithm, we can calculate $C_{n}^{2}$ in the height range of Δh~2Δh, that is, $C_{n2}^{2}$. In this way, the $C_{n}^{2}$ profile of the entire detection path can be obtained.

**Figure 7:**
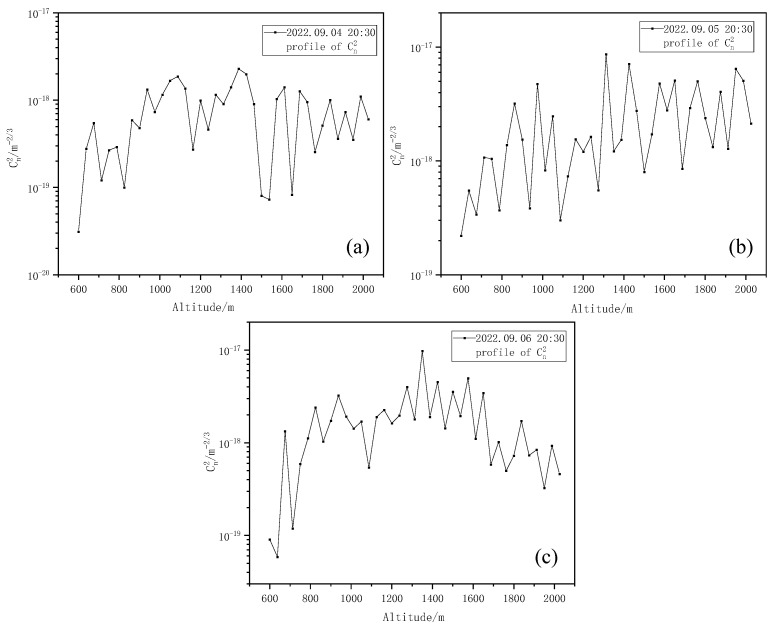
Kolmogorov turbulence intensity $C_{n}^{2}$ profiles. (**a**) Kolmogorov turbulence intensity $C_{n}^{2}$ profiles at 20:30 on 4 September 2022. (**b**) Kolmogorov turbulence intensity $C_{n}^{2}$ profiles at 20:30 on 5 September 2022. (**c**) Kolmogorov turbulence intensity $C_{n}^{2}$ profiles at 20:30 on 6 September 2022.

Figure 7 shows the Kolmogorov turbulence $C_{n}^{2}$ profiles at three different times obtained using the stratified iterative inversion algorithm combined with the scintillation index, as shown in Figure 6.

**Figure 8:**
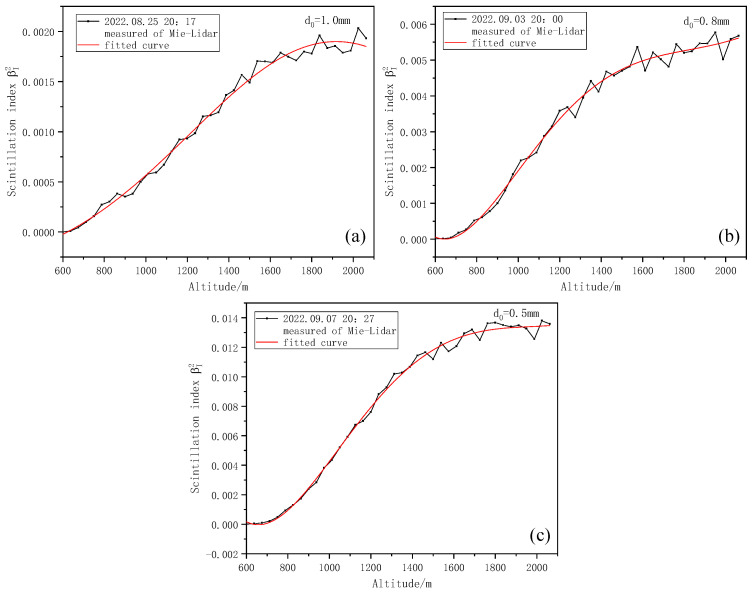
Scintillation index with height for different iris diaphragm diameters. (**a**) Variation of scintillation index with height when iris diaphragm diameter is 1.0 mm. (**b**) Variation of scintillation index with height when iris diaphragm diameter is 0.8 mm. (**c**) Variation of scintillation index with height when iris diaphragm diameter is 0.5 mm.

**Figure 9:**
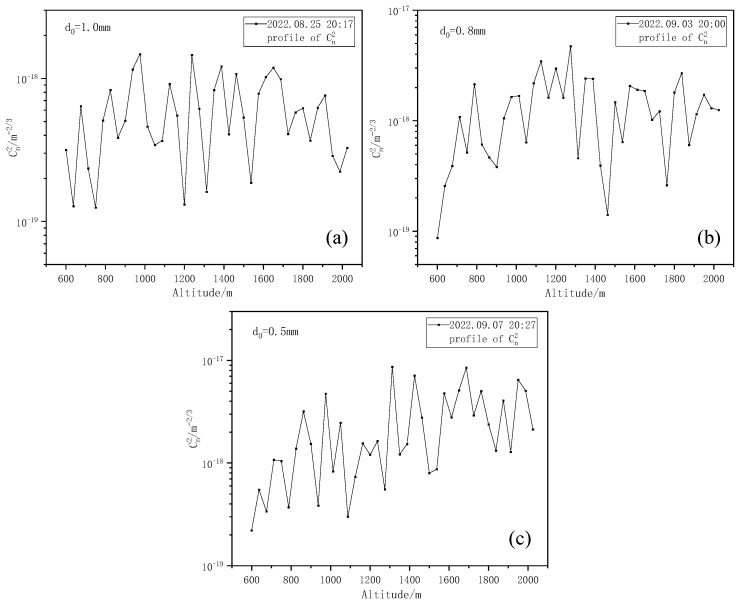
Kolmogorov turbulence intensity $C_{n}^{2}$ profiles with different iris diaphragm diameters. (**a**) Kolmogorov turbulence intensity $C_{n}^{2}$ profiles when iris diaphragm diameter is 1.0 mm. (**b**) Kolmogorov turbulence intensity $C_{n}^{2}$ profiles when iris diaphragm diameter is 0.8 mm. (**c**) Kolmogorov turbulence intensity $C_{n}^{2}$ profiles when iris diaphragm diameter is 0.5 mm.

As can be seen from Figure 7, the Kolmogorov turbulence intensity $C_{n}^{2}$ profile obtained from inversion presents fluctuation changes with an increase in altitude, and the intensity of the upper air turbulence is small, belonging to weak turbulence [35]. Within the detection range, a turbulent layer with relatively weak turbulence intensity appears at a height of 600~800 m. Subsequently, the turbulence intensity increases with the increase in height and gradually becomes stable, which is more consistent with the general change law of turbulence intensity with height. Then, we change the iris diaphragm diameter $d_{0}$ to detect again. Here, three iris diaphragm sizes are selected for comparison: the iris diaphragm diameters $d_{0}$ are 1.0 mm, 0.8 mm, and 0.5 mm, respectively. The variation in the corresponding scintillation index and Kolmogorov turbulence intensity $C_{n}^{2}$ profiles are shown in Figure 8 and Figure 9, respectively.

As can be seen from Figure 8, the smaller the iris diaphragm diameter $d_{0}$, the greater the resulting scintillation index. We analyzed the cause of this situation and found that, due to the existence of the aperture-smoothing effect, the area of the aperture leads to the generation of uncorrelated regions of light intensity fluctuation, and the total scintillation will be weakened due to the cancellation of uncorrelated elements, finally resulting in a small scintillation index. From Figure 9, the smaller the iris diaphragm diameter $d_{0}$, the greater the Kolmogorov turbulence intensity obtained by inversion.

**Figure 10:**
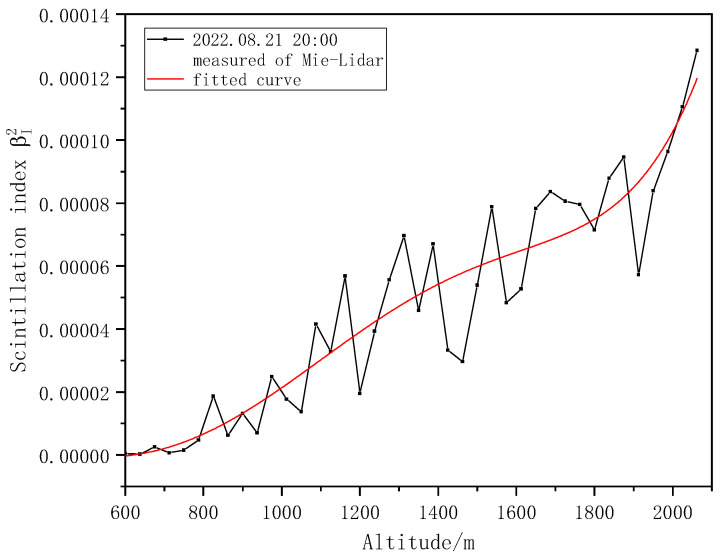
Variation in scintillation index with height on cloudy days.

In the experimental observation, the atmospheric turbulence intensity profile under different weather conditions was also detected. On 21 August 2022, the weather changed from overcast to light rain, with thick clouds in the sky and low temperatures. The diameter of the iris diaphragm is $d_{0}$ = 0.8 mm, and the variation in the scintillation index detected with height is shown in Figure 10.

**Figure 11:**
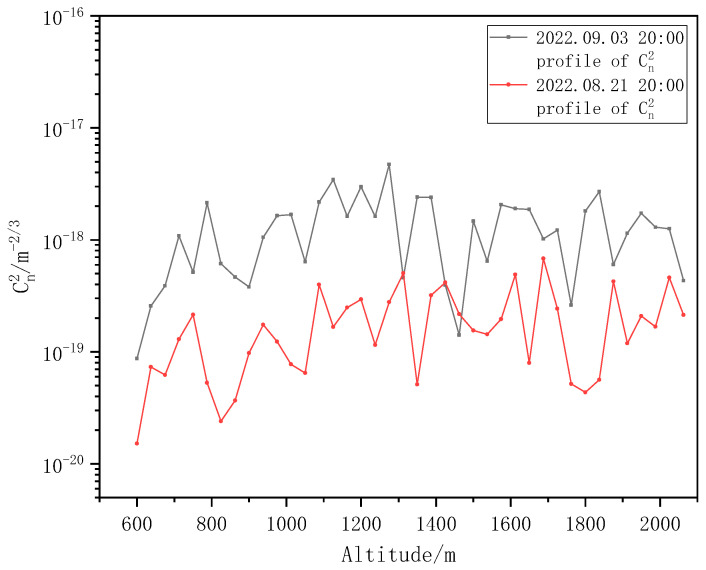
Kolmogorov turbulence intensity profiles under different weather conditions.

The Kolmogorov turbulence intensity $C_{n}^{2}$ profiles corresponding to the cloudy day on August 21 and the sunny day on September 3 were compared, as shown in Figure 11.

It is clear that the scintillation index and turbulence intensity under cloudy conditions are significantly lower than those under sunny conditions, and the Kolmogorov turbulence intensity on cloudy days is approximately one order of magnitude smaller than that on sunny days. This is because the random fluctuation in atmospheric temperature is the main cause of the random fluctuation in the atmospheric refractive index, the temperature fluctuation on cloudy days is lower than that on sunny days, and the temperature fluctuation is less obvious than that on sunny days. On sunny days, solar radiation keeps heating the surface during the daytime, and the heat keeps transmitting upward, resulting in strong temperature fluctuations and a significant turbulence effect.

#### 3.2.2. Non-Kolmogorov Turbulence Detection

Some research shows that for the atmospheric measurements of the tropopause, stratosphere, and near the ground, the turbulence structure-function or turbulence spectrum obtained in experiments still has power law characteristics, but the power law value deviates from –11/3 of the Kolmogorov turbulence spectrum in most cases [36,37]. Therefore, non-Kolmogorov turbulence is defined. The Kolmogorov turbulence three-dimensional power spectral density can be expressed as [2]

(7) $$\Phi_{n}\left(\kappa \right)=0.033C_{n}^{2}\kappa^{-\frac{11}{3}},\frac{1}{L_{0}}\leq \kappa \leq \frac{1}{l_{0}}$$

where $L_{0}$ is the outer scale of turbulence; $l_{0}$ is the inner scale of turbulence, and κ is the space wave number. Meanwhile, the non-Kolmogorov turbulence three-dimensional power spectral density has similar expressions with the Kolmogorov turbulence three-dimensional power spectral density [38]:

(8) $$\Phi_{n}\left(\kappa ,\alpha \right)=A\left(\alpha \right)\tilde{C_{n}^{2}}\kappa^{-\alpha },\;\;\frac{1}{L_{0}}\leq \kappa \leq \frac{1}{l_{0}}$$

where α is the scale index of the three-dimensional power spectrum, also known as the spectral power law, and $\tilde{C_{n}^{2}}$ is the atmospheric refractive index structure constant corresponding to the spectral power rate α within a certain range, which is called the generalized refractive index structure constant, and the unit is $m^{3-\alpha }$. A(α) is called the consistency function, and its function is to make the structure function and power spectrum of the scale index α in a certain range interchangeable, as follows [36]:

(9) $$A\left(\alpha \right)=\frac{\Gamma \left(\alpha -1\right)}{4\pi^{2}}\cos\left(\frac{\alpha \pi }{2}\right),3<\alpha <5$$

For non-Kolmogorov turbulence, under weak fluctuation conditions, the relationship between the variance in normalized light intensity fluctuation (scintillation index) and the variance in logarithmic amplitude fluctuation still satisfies Equation (3). However, the variance in the logarithmic amplitude fluctuation in spherical waves under non-Kolmogorov turbulence is no longer as is shown in Equation (4) but is turned into the following form [39]:

(10) $$\sigma_{\chi }^{2}\left(\alpha \right)=0.033k^{\frac{7}{6}}\int_{0}^{L}\tilde{C_{n}^{2}}\left(h\right)B\left(\alpha \right){\left[\sec\left(\theta \right)\right]}^{\frac{\alpha }{2}}{\left(1-\frac{z}{L}\right)}^{\frac{\alpha -2}{2}}z^{\frac{5}{6}}dz,3<\alpha <5$$

(11) $$B\left(\alpha \right)=\frac{-\pi^{3}}{2\Gamma \left(\frac{\alpha }{2}\right)\cos\left(\frac{\pi \alpha }{4}\right)}$$

where θ is the zenith angle, and the detection path here is the vertical path, so θ = 0. Therefore, the scintillation index of non-Kolmogorov turbulence is written as:

(12) $$\beta_{I}^{2}\left(\alpha \right)=\exp\left(4\sigma_{\chi }^{2}\left(\alpha \right)\right)-1\approx 4\sigma_{\chi }^{2}\left(\alpha \right)$$

**Figure 12:**
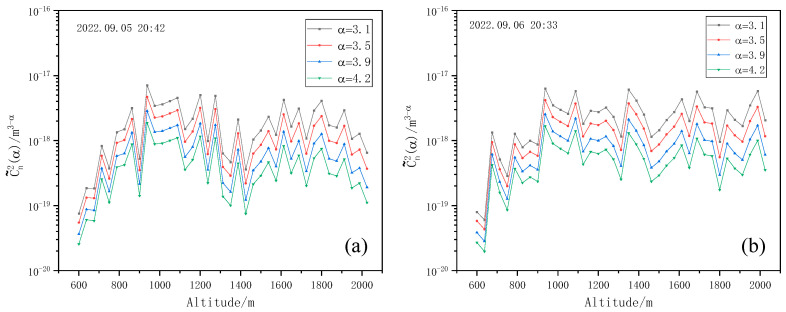
Non-Kolmogorov turbulence intensity profiles with different spectral power laws, α. (**a**) Non-Kolmogorov turbulence intensity profiles with different spectral power laws, α at 20:42 on 5 September 2022. (**b**) Non-Kolmogorov turbulence intensity profiles with different spectral power laws, α at 20:33 on 6 September 2022.

The detection method and condition for the non-Kolmogorov turbulence profile are the same as that for Kolmogorov turbulence. The laser light source is 532 nm, the emission frequency is 10 Hz, and the iris diaphragm diameter $d_{0}$ is 0.5 mm. Here, the non-Kolmogorov turbulence profiles at two different time periods are calculated by Newton iteration inversion, and four different values of spectral power law α are selected as 3.1, 3.5, 3.9 and 4.2, respectively. The result is shown in Figure 12.

It is clear that the larger the value of the spectral power law, α, the smaller ${\tilde{C}}_{n}^{2}$ is. In the actual measurement, due to the anisotropy of turbulence, the common influence of various spectral power laws should be considered to obtain the final ${\tilde{C}}_{n}^{2}$ profile.

### 3.3. Verification

Since there is no synchronous-sounding data for comparison, we use the atmospheric turbulence profile model for comparison and verification. The most commonly used model is the Hufnagel–Valley (HV) model, which allows for changes in the upper air speed and near-ground turbulence levels to be applied to atmospheric conditions at different locations. The model is given by [40]

(13) $$C_{n}^{2}\left(h\right)=5.94\times 10^{-53}h^{10}e^{-\frac{h}{1000}}{\left(\frac{v}{27}\right)}^{2}+2.7\times 10^{-16}e^{-\frac{h}{1500}}+Ae^{-\frac{h}{100}}$$

where v is the wind speed in the upper air; and A denotes $C_{n}^{2}$ in the near-surface boundary layer. The most commonly used value is v = 21 m/s and A = 1.7 × 10^−14^ m^−2/3^, so it is also called the HV-21 model.

The HV model is modified to conform to the night-time meteorological conditions of the Yinchuan area. In Equation (13), the first term represents the strong turbulent layer conditions that often occur in the tropopause, the second term represents the turbulent layer conditions in the boundary layer, and the third term represents the turbulent layer conditions in the free atmosphere [41]. According to our inquiry of relevant data and the data of the National Meteorological Center, the wind speed during the experiment was about 2 m/s [42]. As for the turbulence in the boundary layer and the free atmosphere, according to the long-term observation results in the laboratory, it is found that the turbulence intensity in the boundary layer in Yinchuan is about 3.02 × 10^−18^ m^−2/3^, and that in the free atmosphere is about 5.01 × 10^−17^ m^−2/3^. Therefore, the $C_{n}^{2}$ profile model conforming to the Yinchuan area is fitted, and the model formula is shown as follows:

(14) $$C_{n}^{2}\left(h\right)=3.26\times 10^{-55}h^{10}e^{-\frac{h}{1000}}+3.02\times 10^{-18}e^{-\frac{h}{1500}}+5.01\times 10^{-17}e^{-\frac{h}{100}}$$

**Figure 13:**
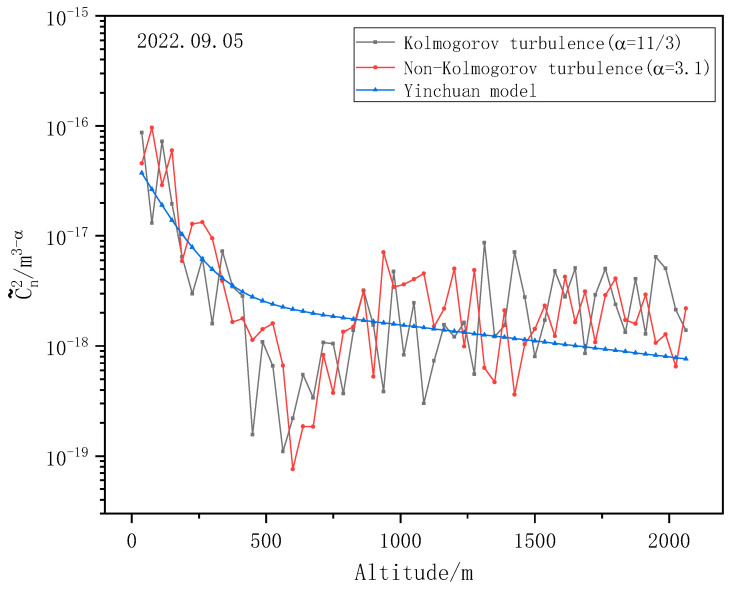
Comparison of atmospheric turbulence profiles detected by Mie-scattering lidar and the HV-night model.

The laser light source was 532 nm, the emission frequency was 10 Hz, and the diameter of the telescope diaphragm $d_{0}$ = 0.5 mm. The day of the experiment was clear and windless. Kolmogorov turbulence intensity and Non-Kolmogorov turbulence intensity were detected, respectively, and the Kolmogorov turbulence and the non-Kolmogorov turbulence profiles were compared with the $C_{n}^{2}$ model of Yinchuan. In order to simplify the comparison process, the spectral power law α = 3.1 was used uniformly to represent non-Kolmogorov turbulence. Figure 13 shows the comparison results.

Regarding the comparison, it can be seen that the overall variation trend of the ${\tilde{C}}_{n}^{2}$ profile detected by Mie-scattering lidar is consistent with the HV-night model in the Yinchuan area, which shows that ${\tilde{C}}_{n}^{2}$ at low altitude decreases with the increase in altitude, while ${\tilde{C}}_{n}^{2}$ at high altitude gradually increases with the increase in altitude and tends to be stable. Both the Kolmogorov turbulence $C_{n}^{2}$ profile and the non-Kolmogorov turbulence ${\tilde{C}}_{n}^{2}$ profile reach their minimum values at approximately 600 m. When compared with the HV-night model of the Yinchuan area, the $C_{n}^{2}$ profile and the ${\tilde{C}}_{n}^{2}$ profile are both one order of magnitude smaller in the low-altitude range, and approximately one order of magnitude larger in the high-altitude range. The actual detection results fluctuate near the theoretical value. The average relative error of Kolmogorov turbulence is 47.07%, and that of non-Kolmogorov turbulence is 52.54%.

This phenomenon has been analyzed in detail, and there are two reasons for this. The first reason is the selection of the HV-night model coefficient. Since there is no real-time high-resolution data, only the meteorological data of that day were used to estimate the wind speed, near-surface turbulence intensity, and boundary layer turbulence intensity, resulting in the difference between the model and the actual detection results. Moreover, due to the overlap of the field of view, the turbulence intensity in the low-altitude range was different from the theoretical value. Another reason is that the turbulence has internal and external scale changes, but the change in the internal and external scales with height was not considered in the inversion process. It is assumed that the inner scale is 0 and the outer scale is infinite for the entire detection path, thus resulting in measurement errors. The $C_{n}^{2}$ profiles of Kolmogorov turbulence and the ${\tilde{C}}_{n}^{2}$ profiles of non-Kolmogorov turbulence obtained by inversion are not entirely accurate. 

However, in general, the detected atmospheric turbulence intensity profile is basically consistent with the trend of the HV-night model, and the detection results are distributed near the theoretical value, indicating that it is feasible for the $C_{n}^{2}$ profile and the ${\tilde{C}}_{n}^{2}$ profile to be detected by Mie-scattering lidar.

## 4. Conclusions

Based on RTS theory, Mie-scattering lidar technology was designed and built for the detection of atmospheric turbulence, and the vertical atmospheric refractive index constant profiles of Kolmogorov turbulence and non-Kolmogorov turbulence were detected and calculated, respectively.

The return light intensity signal on the vertical path was analyzed statistically, and the actual probability distribution of the light intensity was fitted. The results show that the actual probability density distribution of logarithmic light intensity on the vertical path roughly obeys the normal distribution under weak fluctuations.

The atmospheric turbulence was classified into Kolmogorov turbulence and non-Kolmogorov turbulence, and the inversion of the $C_{n}^{2}$ profile was carried out. For Kolmogorov turbulence, the scintillation index profile was obtained by a return signal, and the $C_{n}^{2}$ profile was obtained by hierarchical iterative inversion. By changing the iris diaphragm diameter, $d_{0}$, it was found that the smaller the iris diaphragm diameter, $d_{0}$, the larger the scintillation index, which is due to the existence of the aperture-smoothing effect. In addition, the atmospheric turbulence intensity under different weather conditions was also compared. The results show that the turbulence intensity under cloudy weather conditions was approximately one order of magnitude smaller than that under sunny weather conditions. For non-Kolmogorov turbulence, the detection method was the same as that for Kolmogorov turbulence; by changing the value of the spectral power law, α, it was found that the larger the value of spectral power law, α, the smaller the intensity of non-Kolmogorov turbulence.

Moreover, the detection results of Mie-scattering lidar were compared with the HV-night model in the Yinchuan area. The results show that the Kolmogorov turbulence and non-Kolmogorov turbulence intensity profiles were consistent with the variation trend of the HV-night model, and the detected turbulence intensity fluctuated around the model. The reasons for this phenomenon can be summarized as two aspects: one is that the parameter selection in the model is not accurate enough, and the other is that the turbulence has internal and external scale changes. However, in general, it is feasible to use Mie-scattering lidar to detect atmospheric turbulence.

In the future, the influence of the internal and external scales of turbulence varying with height should be taken into account, and more accurate and applicable inversion algorithms for the $C_{n}^{2}$ profile should be selected to further improve the inversion accuracy from the scintillation index to the structure constant of the atmospheric refractive index $C_{n}^{2}$.

## Figures and Tables

**Table 1:** Parameters of atmospheric turbulence detection Mie-scattering lidar.

System Name	Technical Parameters
**Laser Emission System**	
Type of laser	Pulsed solid-state laser (Nd:YAG)
Operating wavelength/nm	532
Repetition rate/Hz	10
Single-pulse energy/mJ	150
Width of pulse/ns	10
Laser divergence angle/mrad	≤0.5
Stability of energy	≤3%
**Receiving Optical System**	
Type of telescope	Schmidt-Cassegrain
Aperture/mm	254
Focal length/mm	2500
Diameter of iris diaphragm/mm	0~12
Coupling fiber length/m	1.5
Optical fiber diameter/μm	800
Numerical aperture N.A.	0.22
Filter/nm	532 ± 0.2
**Signal Acquisition System**	
Photomultiplier tube model	R9880U
Diameter of photosensitive surface/mm	8
Model of oscilloscope	DPO410B
Bandwidth/GHz	1
Frequency of sampling/M/s	100

## Data Availability

The relevent data used to support the findings of this study are available from the corresponding author upon request.
